# Advances in Antibacterial, Anti‐Biofouling, and Corrosion‐Resistant Surface Technologies

**DOI:** 10.1002/cbin.70140

**Published:** 2026-01-27

**Authors:** Anca Mazare, Wolfgang H. Goldmann, Alexander B. Tesler

**Affiliations:** ^1^ Department of Materials Science and Engineering Institute for Surface Science and Corrosion, Faculty of Engineering, Friedrich‐Alexander‐Universität Erlangen‐Nürnberg Erlangen Germany; ^2^ Department of Physics Biophysics Group, Friedrich‐Alexander‐Universität Erlangen‐Nürnberg Erlangen Germany; ^3^ Department of Materials Engineering, Faculty of Engineering Bar‐Ilan University Ramat‐Gan Israel

## Abstract

This commentary provides a comprehensive and forward‐looking analysis of biofouling and the development of antifouling technologies. It culminates in two innovative, non‐toxic approaches: aerophilic surfaces and liquid‐infused slippery surfaces (LISS/SLIPS).

To date, many papers have been published that present a compelling and diverse array of research advancements aimed at addressing critical issues such as biofouling, antibacterial properties, and the corrosion of various materials, particularly in aquatic environments and biomedical applications. Ensuring material durability and preventing biological interactions in subaqueous contexts is an increasingly pertinent challenge, given the expanding use of metals and composites in marine engineering and medical devices. These studies offer unique insights into surface engineering and material science, and their applications for improving the performance of various substrates (Goldmann 2021).

The “One‐Pot Universal Fabrication of Slippery Surfaces” refers to a process that allows for the creation of surfaces that repel liquids and reduce friction in a single‐step, efficient method. This versatile method has paved the way for broader implementation in different applications, showing that efficiency and versatility can be achieved without compromising performance (Tesler et al. 2021). In response to the demand for eco‐friendly solutions, a study entitled “Nontoxic Liquid‐Infused Slippery Coatings on Steel Substrates Inhibit Corrosion and Biofouling Adhesion” showed that liquid‐infused siloxane‐based coatings can efficiently prevent biofouling and corrosion on steel surfaces. This study highlighted the non‐toxic nature of the formulation, aligning with the growing demand for sustainable materials in regulatory circles (Tesler et al. 2022).

A more recent investigation outlined a methodology for the fabrication of slippery, anti‐fouling, and corrosion‐resistant coatings for aluminum (Prado et al. 2023). The covalent binding of the silicone lubricant ensures the longevity of the surface properties, which could have significant implications for marine and industrial applications. Further investigations examined UV‐grafted polydimethylsiloxane coatings and demonstrated how surface modifications can improve the corrosion resistance of zinc substrates. Zinc's inherent biodegradability aligns with the principles of eco‐friendly initiatives, making this combination particularly appealing for temporary applications in the biomedical field (Pupillo et al. 2024).

Subsequent research assessed the stability and longevity of aerophilic surfaces, i.e., a special type of superhydrophobic surface, under prolonged sub‐aquatic conditions. The findings provided critical insights into the durability and maintenance of such coatings for developing reliable anti‐corrosive technologies (Tesler et al. 2023). These studies have presented a theoretical framework for predicting the stability of plastron, defined as a gas layer that maintains superhydrophobicity in the face of environmental stresses, particularly in underwater applications (Tesler et al. 2024). Other studies highlighted the potential of aerophilic surfaces to provide corrosion resistance to metals submerged in water. These surfaces maintain a stable air layer that inhibits water contact, thus mitigating oxidation processes and enhancing material longevity. Researchers focused on the adhesion dynamics of fluorinated phosphate esters on metal oxides and provided key insights into developing underwater aerophilic surfaces. Understanding the binding kinetics is essential for creating surfaces that retain their properties and functionality in aquatic environments (Mazare, Ulubas, et al. 2025).

Furthermore, a novel coating has been developed that uses ultra‐slippery surfaces to reduce biofouling. The findings indicated that these surfaces' inherent low‐friction characteristics can markedly reduce marine organism attachment, thereby providing sustainable alternatives to conventional biofouling remedies. Additionally, a number of researchers have explored the dual function of metal‐organic frameworks (MOFs) integrated with silver for antibacterial mechanisms (Mazare, Goldmann, et al. 2025). This research study highlighted the synergistic effects of titania's photocatalytic properties in conjunction with the release of silver ions. The findings suggest a promising avenue for developing antimicrobial surfaces for environmental and biomedical applications.

In summary, these papers collectively advance our understanding of, and technological capabilities in, creating surfaces that resist biofouling and corrosion while exhibiting antibacterial behavior. The integration of novel materials and surface engineering techniques has emerged as a promising solution to the challenges posed by real‐world marine and medical applications. It contributes to the development of sustainable, resilient materials for the future.

The emphasis on improving mechanical properties in conjunction with antibacterial performance exemplifies a holistic approach to material design, particularly for implantable devices. These studies underscore the significance of durability, ecology, and advanced functionality in the development of future innovations. The integration of advanced materials and coatings with surface engineering is not merely a response to current needs; it is also an effort to develop robust, sustainable solutions for harsh marine and medical environments. The translation of these findings into practical applications that can address some of today's most pressing challenges in a sustainable manner is dependent on ongoing collaboration among chemists, engineers, and biologists (Tesler and Goldmann 2025).

## Data Availability

The data that support the findings of this study are available on request from the corresponding author. The data are not publicly available due to privacy or ethical restrictions.
